# Quality of Life in Thyroid Cancer Patients

**DOI:** 10.7759/cureus.45222

**Published:** 2023-09-14

**Authors:** Jolan S Alsaud, Mariam S Alharbi, Abdullah I Aldekhail, Rakan A Almesned, Fatimah S Alsultan, Meshari Alharbi, Hamad Alshubrumi

**Affiliations:** 1 College of Medicine, Qassim University, Buraydah, SAU; 2 Endocrinology and Diabetes Center, King Fahad Specialist Hospital, Buraydah, SAU

**Keywords:** quality improvement, thyroid cancer patients, saudi arabia, thyroid cancer, quality of life

## Abstract

Thyroid cancer ranks as the ninth most common cancer worldwide and third in Saudi Arabia. Given thyroid cancer's high incidence, thyroid cancer patients' quality of life (QoL) has been a concern for many years. However, no study has been dedicated to assessing thyroid cancer patients' QoL in Saudi Arabia. Thus, we aimed to fill this gap by assessing thyroid cancer patients' QoL in Saudi Arabia. This cross-sectional study was conducted among thyroid cancer patients attending outpatient clinics at the Diabetes and Endocrinology Centre, King Fahad Specialist Hospital, Qassim region, Saudi Arabia, from 2017 to 2023. The European Organization for the Research and Treatment of Cancer Quality of Life Questionnaire C30 (EORTC QLQ-C30) assessed the patients' QoL through an individual interviewer-administered technique. We used RStudio to perform statistical analysis. Statistical differences between groups based on QoL scores were assessed using the Wilcoxon and Kruskal-Wallis rank-sum tests. Our results showed that for QoL scores of functional domains, the lowest was in social functioning, and the highest was in cognitive functioning. Regarding the subscales of symptoms, the highest scores were fatigue and insomnia. In conclusion, numerous factors affect thyroid cancer patients' QoL. Recognition and appropriate management of these factors will improve the overall QoL; there is a need to understand what is driving these factors in further clinical studies.

## Introduction

The World Health Organization (WHO) defines quality of life (QoL) as "individuals' perceptions of their position in life in the context of the culture and value systems in which they live and in relation to their goals, expectations, standards, and concerns" [1]. The literature interchangeably uses the terms health-related quality of life (HRQoL) and QoL. However, several attempts exist to differentiate between these terms to create one general definition and another specific one focusing more on health-related QoL [2]. Moreover, QoL is a multidimensional construct that includes physical, social, psychological, disease, and treatment-related symptoms. One of the proposed and widely accepted definitions of QoL in cancer studies defines it as "a state of well-being which is a composite of two components: 1) the ability to perform everyday activities that reflect physical, psychological, and social well-being and 2) patient satisfaction with levels of functioning and the control of disease and/or treatment-related symptoms" [3]. The first component of the state of well-being indicates a more objective aspect of the QoL. By contrast, the second component is more subjective and relies on the patient's reported satisfaction. The Centers for Disease Control and Prevention (CDC) has defined HRQoL as "an individual's or group's perceived physical and mental health over time." Measuring the HRQoL proved its importance and usefulness for assessing the efficacy of treatments. Measuring the HRQoL helps reveal the burden of diseases and explore the relationships between HRQoL and different risk factors. Tracking the HRQoL can also help in monitoring the progress/regress of a country in achieving its health objectives [4]. Measuring the HRQoL is a method that can reflect multiple clinical indicators of the patients' health. In addition, the goal of healthcare services should be addressing more than just the results of labs and imaging studies. Instead, it must also address the patients' general well-being and assess their satisfaction level.

Thyroid cancer is one of the most common cancers globally. According to the latest Global Cancer Observatory (GLOBOCAN) estimates, thyroid cancer was the ninth most commonly diagnosed worldwide, with an incidence of 586,202 cases [5]. In the United States and other industrialized countries, the incidence of thyroid cancer has been increasing for the past 30 years [6-9]. The leading causes behind the increase in the incidence of thyroid cancer are the improvement of medical imaging and ionizing radiation, environmental and genetic factors, and better accessibility of the population to healthcare services [10]. In Saudi Arabia, the latest cancer incidence report was issued by the Saudi Health Council in 2018, showing that thyroid cancer is the third most common cancer among Saudi nationals after breast and colorectal cancers. Thyroid cancer in Saudi Arabia ranked second among women and 10th among men. Furthermore, the report showed that the annual incidence of thyroid cancer among Saudi nationals is 1,323 cases. In Saudi Arabia, thyroid cancer's age-standardized incidence rate (ASR) was 10.6/100,000 for women and 2.9/100,000 for men [11]. The high incidence of thyroid cancer patients necessitated building infrastructure to meet the needs of affected patients. Among the infrastructure that has been built is the center we collected data from for this study, the Diabetes and Endocrinology Centre at King Fahad Specialist Hospital in Qassim region. Most thyroid cancers fall into these categories: papillary thyroid carcinoma (PTC) and follicular thyroid carcinoma (FTC), both of which are further classified as differentiated thyroid carcinoma (DTC), undifferentiated thyroid carcinoma (UTC), and medullary thyroid carcinoma (MTC) [12]. Given thyroid cancer's high incidence worldwide, the QoL of thyroid cancer patients has been an area of concern for many years [10].

Literature review

Multiple instruments have been developed to measure QoL among cancer patients [13]. The European Organization for the Research and Treatment of Cancer (EORTC) developed one commonly used instrument named the Core Quality-of-Life Questionnaire (QLQ-C30). It was developed to assess the QoL of cancer patients, and due to the comprehensiveness of this instrument and the fact that it has been translated and validated into over 100 languages, this instrument was widely used by thousands of studies [14]. The QLQ-C30 was used in many studies to assess the QoL in thyroid cancer patients [15-17]. A group of researchers analyzed the HRQoL among patients with DTC at the Medical University of Innsbruck using the QLQ-C30; they found that the patients' HRQoL was worse than the general population in all domains except the cognitive functioning, diarrhea, and financial impacts. Differences between DTC patients and the general population in HRQoL were particularly significant for role functioning and fatigue [15]. In Shandong Provincial Qianfoshan Hospital, patients with DTC undergoing radioactive iodine-131 treatment were recruited. Their QoL was measured using QLQ-C30, and after one year of psychological and behavioral interventions, the patients' QoL was significantly improved [16].

Interestingly, a South Korean study showed that even disease-free survivors of DTC had lower HRQoL when compared to the general population [17]. A UK-based study assessing the QoL among DTC patients using the European Quality of Life 5 Dimensions revealed that thyroid cancer patients had lower QoL than colorectal cancer patients. Studying patients with a type of thyroid cancer more invasive than DTC could yield worse QoL results [18]. However, it is worth mentioning that thyroid cancer patients have better survival compared to colorectal cancer patients [19].

In Saudi Arabia, multiple studies have been conducted to assess the QoL among cancer patients attending the outpatient clinics of various tertiary hospitals [20-22]. However, no study is dedicated to assessing the QoL among thyroid cancer patients despite the previously discussed significance of assessing the QoL [4]. We believe that assessing the QoL among thyroid cancer patients can give an insight into the quality of healthcare provided them. We aimed to fill this gap by assessing the QoL among thyroid cancer patients attending outpatient clinics at the Diabetes and Endocrinology Centre, King Fahad Specialist Hospital, Qassim region, Saudi Arabia.

## Materials and methods

Study design, area, population, and duration

This cross-sectional study was conducted at the Diabetes and Endocrinology Centre, King Fahad Specialist Hospital, Qassim region, Saudi Arabia. All thyroid cancer patients attending outpatient clinics from 2017 to 2023 who agreed to participate in the study were included.

Sample size and selection of samples

We reached a total of 129 patients. Our required minimal sample size was 124 patients calculated using Cochran’s equation together with a population correction, where the population size is 181 patients based on the number of thyroid cancer patients seen at the Diabetes and Endocrinology Centre, King Fahad Specialist Hospital, Qassim region, Saudi Arabia, from 2017 to 2023, the confidence level is 95%, the precision level is ±5%, and the estimated proportion is 0.5.

The sampling technique is a non-probability convenient sampling technique. All thyroid cancer patients attending the outpatient clinics from 2017 to 2023 who agreed to participate in the study were included.

Tools and data collection methods

A retrospective chart review of thyroid cancer patients seen at the Diabetes and Endocrinology Centre, King Fahad Specialist Hospital, Qassim region, Saudi Arabia, from 2017 to 2023 was done. Contact information was obtained from the center's inpatient medical files. The European Organization for the Research and Treatment of Cancer Quality of Life Questionnaire C30 (EORTC QLQ-C30) [23] was used to assess the QoL through an individual interviewer-administered technique. Data collection was done from November 2022 to April 2023. The survey consisted of 30 items, which assess five functional subscales (physical, cognitive, role, social, and emotional) and nine symptomatic subscales (fatigue, nausea/vomiting, dyspnea, insomnia, pain, appetite loss, constipation, diarrhea, and financial difficulties) and a global health status (GHS) subscale. All the subscales (except the GHS subscale) were collected on a four-point Likert scale ranging between 1 = “not at all” to 4 = “very much.” Items of the GHS subscale were recorded on a seven-point scale from 1 = “very poor” to 7 = “excellent.” The scores of the symptoms’ subscales were reversed, and an overall QoL score was computed by summing up the items of the functional subscales and symptomatic subscales (except financial difficulties) [24]. Therefore, a higher QoL score indicated a higher QoL indicator. All the scores were converted to their percentage counterparts to be easily interpreted. The questionnaire used was the Arabic version of the EORTC QLQ-C30 developed, translated, and validated by the EORTC.

Data management and analysis plan

We used RStudio (R version 4.3.0) to perform statistical analysis. We used frequencies and percentages to express categorical variables, whereas continuous variables were presented as the median and interquartile range (IQR). Statistical differences between different groups based on QoL scores were assessed using a Wilcoxon rank-sum test for variables with two groups and a Kruskal-Wallis rank sum test for variables with three or more categories. Statistical significance was considered at p-value < 0.05.

Ethical considerations

An informed consent explaining the aim of the study and how the data will be managed was obtained from each participant. Only the researchers can access the collected data. We obtained ethical approval from the Regional Research Ethics Committee (NCBE, No. H-04-Q-001) for the application and publication of the study (protocol code 607-44-3752; date of approval: October 10, 2022).

## Results

Demographic characteristics of patients

Data from 129 patients with thyroid cancer were analyzed in the current study. The majority of patients were women (86.8%), unemployed/housewives (70.5%), married (79.8%), and aged more than 35 years (80.6%) (Table 1).

**Table 1:** Demographic characteristics of the patients (n = 129)

Parameter	Category	Number (%)
Gender	Male	17 (13.2%)
	Female	112 (86.8%)
Age	18 to 35	25 (19.4%)
	36 to 50	52 (40.3%)
	>50	52 (40.3%)
Occupation	Government employee	29 (22.5%)
	Private employee	9 (7.0%)
	Unemployed/housewife	91 (70.5%)
Marital status	Single	15 (11.6%)
	Married	103 (79.8%)
	Divorced/widowed	11 (8.5%)

Description of the QoL scores

Descriptive statistics for the QoL scores are provided in Table 2. For the QoL scores of functional domains, the lowest score was relevant to social functioning (median = 25.0, IQR = 25.0 to 50.0), whereas the highest score was related to cognitive functioning (median = 50.0, IQR = 37.5 to 62.5). Regarding the subscales of symptoms, the highest scores (indicating more discomfort and suffering) were reported for fatigue (median = 50.0, IQR = 33.3 to 75.0) and insomnia (median = 50.0, IQR = 25.0 to 100.0; Table 2).

**Table 2:** Descriptive analysis of the QoL scores IQR: interquartile range, SD: standard deviation, Min: minimum, Max: maximum

Scale	Items	Median (IQR)	Mean ± SD	Min - Max
Functional scales				
Physical functioning	5	40.0 (25.0 to 55.0)	42.1 ± 17.7	25.0 - 85.0
Role functioning	2	37.5 (25.0 to 62.5)	45.8 ± 24.9	25.0 - 100.0
Emotional functioning	4	43.8 (31.3 to 62.5)	50.0 ± 20.8	25.0 - 100.0
Cognitive functioning	2	50.0 (37.5 to 62.5)	52.8 ± 22.4	25.0 - 100.0
Social functioning	2	25.0 (25.0 to 50.0)	41.0 ± 23.6	25.0 - 100.0
Symptoms scales/Items				
Fatigue	3	50.0 (33.3 to 75.0)	52.5 ± 23.0	25.0 - 100.0
Nausea/vomiting	2	25.0 (25.0 to 37.5)	35.8 ± 17.1	25.0 - 100.0
Pain	2	37.5 (25.0 to 62.5)	47.6 ± 23.1	25.0 - 100.0
Dyspnea	1	25.0 (25.0 to 75.0)	49.0 ± 28.4	25.0 - 100.0
Insomnia	1	50.0 (25.0 to 100.0)	60.3 ± 30.2	25.0 - 100.0
Appetite loss	1	25.0 (25.0 to 50.0)	45.5 ± 26.6	25.0 - 100.0
Constipation	1	25.0 (25.0 to 50.0)	43.2 ± 25.0	25.0 - 100.0
Diarrhea	1	25.0 (25.0 to 25.0)	32.9 ± 16.5	25.0 - 100.0
Financial difficulties	1	25.0 (25.0 to 25.0)	34.1 ± 20.5	25.0 - 100.0
Global health status	2	85.7 (71.4 to 100.0)	81.3 ± 19.8	14.3 - 100.0
Overall score	27	60.2 (56.5 to 63.0)	60.5 ± 5.4	49.1 - 76.9

Factors associated with QoL scores

The QoL pain scores were higher among women (median = 43.8, IQR = 25.0 to 62.5) than men (median = 25.0, IQR = 25.0 to 37.5, p = 0.019; Table 3). There were no significant differences in QoL scores based on the different categories of age (Table 4).

**Table 3:** Differences in the QoL scores regarding patients’ gender (n = 129) IQR: interquartile range, n: number, *: significant difference (p-value < 0.05)

Parameter	Men, n = 17 Median (IQR)	Women, n = 112 Median (IQR)	p-value *
Functional scales			
Physical functioning	35.0 (25.0 to 45.0)	40.0 (25.0 to 55.0)	0.377
Role functioning	25.0 (25.0 to 50.0)	37.5 (25.0 to 62.5)	0.180
Emotional functioning	43.8 (37.5 to 56.3)	43.8 (31.3 to 64.1)	0.750
Cognitive functioning	50.0 (50.0 to 62.5)	50.0 (37.5 to 62.5)	0.755
Social functioning	25.0 (25.0 to 37.5)	25.0 (25.0 to 50.0)	0.782
Symptoms scales/items			
Fatigue	41.7 (33.3 to 58.3)	50.0 (31.3 to 75.0)	0.693
Nausea/vomiting	25.0 (25.0 to 37.5)	25.0 (25.0 to 37.5)	0.761
Pain	25.0 (25.0 to 37.5)	43.8 (25.0 to 62.5)	0.019 *
Dyspnea	25.0 (25.0 to 50.0)	50.0 (25.0 to 75.0)	0.053
Insomnia	50.0 (25.0 to 75.0)	62.5 (25.0 to 100.0)	0.389
Appetite loss	50.0 (25.0 to 75.0)	25.0 (25.0 to 50.0)	0.170
Constipation	50.0 (25.0 to 75.0)	25.0 (25.0 to 50.0)	0.335
Diarrhea	25.0 (25.0 to 25.0)	25.0 (25.0 to 25.0)	0.519
Financial difficulties	25.0 (25.0 to 50.0)	25.0 (25.0 to 25.0)	0.156
Global health status	85.7 (85.7 to 100.0)	85.7 (71.4 to 100.0)	0.175
Overall score	60.2 (59.3 to 63.0)	59.7 (56.5 to 63.9)	0.638

**Table 4:** Differences in the QoL scores regarding patients’ age (n = 129) IQR: interquartile range, N: number, *: significant difference (p-value < 0.05)

Parameter	18 to 35, n = 25 Median (IQR)	36 to 50, n = 52 Median (IQR)	> 50, n = 52 Median (IQR)	p-value *
Functional scales				
Physical functioning	30.0 (25.0 to 55.0)	35.0 (28.8 to 50.0)	40.0 (25.0 to 56.3)	0.321
Role functioning	25.0 (25.0 to 50.0)	43.8 (25.0 to 62.5)	37.5 (25.0 to 75.0)	0.393
Emotional functioning	43.8 (31.3 to 62.5)	43.8 (31.3 to 75.0)	43.8 (37.5 to 56.3)	0.858
Cognitive functioning	50.0 (37.5 to 62.5)	50.0 (34.4 to 75.0)	50.0 (37.5 to 62.5)	0.815
Social functioning	25.0 (25.0 to 50.0)	31.3 (25.0 to 62.5)	25.0 (25.0, 40.6)	0.137
Symptoms scales/Items				
Fatigue	41.7 (25.0 to 75.0)	50.0 (31.3 to 75.0)	50.0 (33.3 to 68.8)	0.589
Nausea / Vomiting	25.0 (25.0 to 37.5)	25.0 (25.0 to 37.5)	25.0 (25.0 to 50.0)	0.368
Pain	37.5 (25.0 to 50.0)	37.5 (25.0 to 62.5)	37.5 (25.0 to 62.5)	0.722
Dyspnea	25.0 (25.0 to 75.0)	50.0 (25.0 to 75.0)	50.0 (25.0 to 75.0)	0.844
Insomnia	50.0 (25.0 to 75.0)	75.0 (25.0 to 100.0)	62.5 (25.0 to 100.0)	0.258
Appetite loss	50.0 (25.0 to 75.0)	25.0 (25.0 to 50.0)	25.0 (25.0 to 50.0)	0.579
Constipation	25.0 (25.0 to 50.0)	25.0 (25.0 to 50.0)	25.0 (25.0 to 50.0)	0.662
Diarrhea	25.0 (25.0 to 25.0)	25.0 (25.0 to 25.0)	25.0 (25.0 to 50.0)	0.075
Financial difficulties	25.0 (25.0 to 50.0)	25.0 (25.0 to 25.0)	25.0 (25.0 to 25.0)	0.486
Global health status	85.7 (71.4 to 100.0)	85.7 (71.4 to 100.0)	85.7 (71.4 to 100.0)	0.738
Overall score	59.3 (58.3 to 62.0)	60.2 (57.2 to 65.7)	59.7 (55.6 to 63.0)	0.391

For the occupational status, there were significant differences between government employees, private employees, and unemployed/housewife participants in terms of the scores of physical functioning (p = 0.021), role functioning (p = 0.035), social functioning (p = 0.026), nausea/vomiting (p = 0.035), pain (p = 0.037), appetite loss (p = 0.009), and the GHS (p = 0.018; Table 5).

**Table 5:** Differences in the QoL scores regarding patients’ occupation (n = 129) IQR: interquartile range, N: number, *: significant difference (p-value < 0.05)

Parameter	Government employee, n = 29 Median (IQR)	Private employee, n = 9 Median (IQR)	Unemployed/housewife, n = 91 Median (IQR)	p-value *
Functional scales				
Physical functioning	30.0 (25.0 to 40.0)	50.0 (40.0 to 60.0)	40.0 (25.0 to 55.0)	0.021 *
Role functioning	25.0 (25.0 to 50.0)	62.5 (37.5 to 87.5)	37.5 (25.0 to 62.5)	0.035 *
Emotional functioning	43.8 (31.3 to 56.3)	62.5 (50.0 to 75.0)	43.8 (31.3 to 62.5)	0.057
Cognitive functioning	50.0 (37.5 to 62.5)	62.5 (50.0 to 62.5)	50.0 (37.5 to 68.8)	0.119
Social functioning	37.5 (25.0 to 75.0)	25.0 (25.0 to 62.5)	25.0 (25.0 to 50.0)	0.026 *
Symptoms scales/items				
Fatigue	41.7 (25.0 to 50.0)	66.7 (41.7 to 83.3)	50.0 (33.3 to 75.0)	0.041 *
Nausea/vomiting	25.0 (25.0 to 25.0)	37.5 (25.0 to 62.5)	25.0 (25.0 to 43.8)	0.035 *
Pain	37.5 (25.0 to 37.5)	50.0 (37.5 to 87.5)	50.0 (25.0 to 62.5)	0.037 *
Dyspnea	25.0 (25.0 to 50.0)	50.0 (25.0 to 100.0)	50.0 (25.0 to 75.0)	0.101
Insomnia	50.0 (25.0 to 75.0)	100.0 (25.0 to 100.0)	75.0 (25.0 to 100.0)	0.428
Appetite loss	25.0 (25.0 to 50.0)	75.0 (50.0 to 100.0)	25.0 (25.0 to 50.0)	0.009 *
Constipation	25.0 (25.0 to 50.0)	50.0 (25.0 to 100.0)	25.0 (25.0 to 50.0)	0.524
Diarrhea	25.0 (25.0 to 25.0)	25.0 (25.0 to 25.0)	25.0 (25.0 to 50.0)	0.206
Financial difficulties	25.0 (25.0 to 25.0)	25.0 (25.0 to 50.0)	25.0 (25.0 to 25.0)	0.080
Global health status	92.9 (85.7 to 100.0)	71.4 (57.1 to 85.7)	85.7 (71.4 to 100.0)	0.018 *
Overall score	61.1 (56.5 to 63.0)	63.0 (59.3 to 65.7)	60.2 (56.5 to 63.0)	0.609

Finally, single participants had significantly higher scores of QoL impairment due to appetite loss (median = 50.0, IQR = 37.5 to 100.0) than married participants (median = 25.0, IQR = 25.0 to 50.0), divorced participants (median = 25.0, IQR = 25.0 to 37.5), and widowed participants (median = 25.0, IQR = 25.0 to 37.5, p = 0.013; Table 6).

**Table 6:** Differences in the QoL scores regarding patients’ marital status (n = 129) IQR: interquartile range, N: number, *: significant difference (p-value < 0.05)

Characteristic	Single, n = 15 Median (IQR)	Married, n = 103 Median (IQR)	Divorced/widowed, n = 11 Median (IQR)	p-value *
Functional scales				
Physical functioning	45.0 (25.0 to 57.5)	35.0 (25.0 to 50.0)	40.0 (30.0 to 57.5)	0.647
Role functioning	37.5 (25.0 to 62.5)	37.5 (25.0 to 62.5)	25.0 (25.0 to 43.8)	0.480
Emotional functioning	50.0 (37.5 to 65.6)	43.8 (31.3 to 62.5)	43.8 (34.4 to 53.1)	0.857
Cognitive functioning	50.0 (50.0 to 62.5)	50.0 (37.5 to 62.5)	50.0 (37.5 to 56.3)	0.556
Social functioning	25.0 (25.0 to 43.8)	25.0 (25.0 to 50.0)	25.0 (25.0 to 31.3)	0.317
Symptoms scales/items				
Fatigue	66.7 (37.5 to 79.2)	50.0 (29.2 to 75.0)	50.0 (41.7 to 75.0)	0.499
Nausea/vomiting	25.0 (25.0 to 43.8)	25.0 (25.0 to 37.5)	25.0 (25.0 to 43.8)	0.973
Pain	50.0 (25.0 to 68.8)	37.5 (25.0 to 62.5)	25.0 (25.0 to 43.8)	0.244
Dyspnea	50.0 (25.0 to 75.0)	50.0 (25.0 to 75.0)	25.0 (25.0 to 50.0)	0.586
Insomnia	50.0 (25.0 to 75.0)	75.0 (25.0 to 100.0)	25.0 (25.0 to 87.5)	0.465
Appetite loss	50.0 (37.5 to 100.0)	25.0 (25.0 to 50.0)	25.0 (25.0 to 37.5)	0.013 *
Constipation	25.0 (25.0 to 75.0)	25.0 (25.0 to 50.0)	50.0 (25.0 to 87.5)	0.391
Diarrhea	25.0 (25.0 to 25.0)	25.0 (25.0 to 37.5)	25.0 (25.0 to 25.0)	0.228
Financial difficulties	25.0 (25.0 to 25.0)	25.0 (25.0 to 25.0)	25.0 (25.0 to 25.0)	0.931
Global health status	85.7 (75.0 to 100.0)	85.7 (71.4 to 100.0)	100.0 (82.1 to 100.0)	0.252
Overall score	59.3 (58.3 to 61.1)	60.2 (56.5 to 63.9)	61.1 (58.8 to 63.9)	0.894

## Discussion

To the best of our knowledge, this study is the first study dedicated to assessing the QoL among thyroid cancer patients in Saudi Arabia. Studies that assessed thyroid cancer patients' QoL are limited. Our sample's demographics align with the demographics of thyroid cancer patients in Saudi Arabia. Women constitute 78.9% of all the new cases of thyroid cancer in Saudi Arabia; in our study, they constitute 86.6% of the sample. We believe that this alignment between the percentage of women with thyroid cancer in Saudi Arabia and the proportion of women in our sample enhances the applicability of this study [11]. Patients with cancer often experience psychological burdens due to heightened stress experienced in different functional domains [24]. In our study, the lowest score was in social functioning, unlike a study conducted in Korea, which showed significantly lower scores in all functional domains among disease-free survivors of DTC, including physical, role, cognitive, emotional, and social. This discrepancy could be explained by different contextual or cultural factors open for research [17].

Moreover, our study showed that the pain scores were higher among women than men, which is consistent with the finding of the paper published by Alshehri et al. [20]. According to their study, women tended to score worse in all domains. However, we acknowledge the potential for sample bias since most of our patients were women. Women, in general, tend to score lower than men in HRQoL scores. Different sociodemographic characteristics could explain the gender differences, most importantly individual income, which was found by a study conducted in the USA to be one of the most impactful factors in creating a difference between men's and women's HRQoL scores [25]. Previous multivariate analyses of the patients' age prove a negative relationship between age and global QoL scores [20,21]. By contrast, our study did not show significant differences in QoL scores based on age categories. However, this could be due to the smaller size of our sample.

Interestingly, regarding patients' marital status, our results showed that single participants had significantly higher levels of QoL impairment due to appetite loss than married participants, which contradicts the study published by Alshehri et al. [20] that showed no significant differences in QoL scores based on patients' marital status. The lower level of emotional support might explain the appetite loss reported more among single participants. Our study noted that regarding occupational status, there were significant differences among government employees, private employees, and unemployed/housewife participants in terms of the scores of physical functioning (median = 30.0, IQR = 25.0 to 40.0; median = 50.0, IQR = 40.0 to 60.0; and median = 40.0, IQR = 25.0 to 55.0, respectively; p = 0.021) and also in other aspects of QoL, such as role functioning and clinical symptoms. This is contrast to a study published three years ago by Alshehri et al. [20], which concluded that no significant difference or relationship was found between QoL and occupational status. Furthermore, a study by Lee et al. [17] noted that patients who were employed during the study showed significantly better role functioning and social functioning than unemployed participants.

Limitations

Our research has some limitations. First, we could not access patients' contact information through a population-based cancer registry. Therefore, we must manually select cancer patients from medical centers' systems for QoL studies. This limited our ability to reach a more extensive sample. Second, the Saudi Cancer Registry published inconsistent epidemiological reports (the last was in 2018) that could help understand the incidence and prevalence of different cancers. However, researchers cannot refer to the Saudi Cancer Registry to know the management plan of each patient. Accordingly, assessing the impact of different management lines on the patient's QoL was difficult. Third, this study is based on an interviewer-administered questionnaire. As a result, interviewer bias cannot be eliminated. Fourth, our study was limited to only the Qassim region. Thus, it may not accurately reflect the QoL of thyroid cancer patients in Saudi Arabia. Therefore, we recommend including future studies assessing patients' QoL with different thyroid cancer subtypes and a larger sample from different locations. Assessing the impact of different management lines done for thyroid cancer patients on their QoL is essential. Outcomes should then be compared to the literature, which could be used to evaluate the quality of the healthcare services provided to thyroid cancer patients.

## Conclusions

Numerous physical and psychological factors might affect the QoL among thyroid cancer patients. The QoL scores of thyroid cancer patients in our study are comparable to scores reported in studies worldwide. These similarities indicate that the standard healthcare service was given to thyroid cancer patients in the Qassim region.

We must carefully consider developing a health approach that effectively treats thyroid cancer patients while minimizing the impact of factors negatively affecting the patient's QoL. Recognition and appropriate management of these factors will improve the overall QoL; there is a need to understand what is driving these factors and support the formulation of effective management and primary prevention strategies. The Saudi Cancer Registry needs to create a mechanism by which researchers can easily access individual cancer data without compromising patients' confidentiality. The cooperation between the Saudi Cancer Registry and researchers is essential to ensure better quantity and quality of cancer research.
